# Implementation of a rapid response team in a large nonprofit Brazilian hospital: improving the quality of emergency care through Plan-Do-Study-Act

**DOI:** 10.5935/0103-507X.20190036

**Published:** 2019

**Authors:** Meire Cavalieri Almeida, Margareth Crisóstomo Portela, Elenir Pereira Paiva, Raquel Rodrigues Guimarães, Wilson Coelho Pereira Neto, Priscila Rodrigues Cardoso, Daniel Angelo de Mattos, Izabela Maria Alvim de Castro Cunha Mendes, Marcus Vinicius Tavares, Guillermo Patrício Ortega Jácome, Guilherme Côrtes Fernandes

**Affiliations:** 1 Departamento de Administração e Planejamento em Saúde, Escola Nacional de Saúde Pública, Fundação Oswaldo Cruz - Rio de Janeiro (RJ), Brasil.; 2 Faculdade de Enfermagem, Universidade Federal de Juiz de Fora - Juiz de Fora (MG), Brasil.; 3 Santa Casa de Misericórdia de Juiz de Fora - Juiz de Fora (MG), Brasil.

**Keywords:** Quality of health care, Hospital care, Emergency medical services, Hospital rapid response team, Quality assurance health care, Organizational innovation, Latin America, Qualidade da assistência à saúde, Assistência hospitalar, Serviços médicos de emergência, Time de respostas rápidas de hospitais, Garantia da qualidade dos cuidados de saúde, Inovação organizacional, América Latina

## Abstract

**Objective:**

To describe the implementation of a rapid response team in a large nonprofit hospital, indicating relevant issues for other initiatives in similar contexts, particularly in Latin America.

**Methods:**

In general terms, the intervention consisted of three major components: (1) a tool to detect aggravation of clinical conditions in general wards; (2) the structuring of a rapid response team to attend to all patients at risk; and (3) the monitoring of indicators regarding the intervention. This work employed four half-year Plan-Do-Study-Act cycles to test and adjust the intervention from January 2013 to December 2014.

**Results:**

Between 2013 and 2014, the rapid response team attended to 2,296 patients. This study showed a nonsignificant reduction in mortality from 8.3% in cycle 1 to 5.0% in cycle 4; however, death rates remained stable in cycles 3 and 4, with frequencies of 5.2% and 5.0%, respectively. Regarding patient flow and continuum of critical care, which is a premise of the rapid response system, there was a reduction in waiting time for intensive care unit beds with a decrease from 45.9% to 19.0% in the frequency of inpatients who could not be admitted immediately after indication (p < 0.001), representing improved patient flow in the hospital. In addition, an increase in the recognition of palliative care patients from 2.8% to 10.3% was noted (p = 0.005).

**Conclusion:**

Implementing a rapid response team in contexts where there are structural restrictions, such as lack of intensive care unit beds, may be very beneficial, but a strategy of adjustment is needed.

## Abstract (pt)

**Objetivo:**

Descrever a implantação de um time de resposta rápida em um grande hospital filantrópico, indicando as questões relevantes para as iniciativas em contextos similares, particularmente na América Latina.

**Métodos:**

Em termos gerais, a intervenção consistiu em três componentes principais: (1) uma ferramenta para detecção de agravamento das condições clínicas nas enfermarias gerais; (2) estruturação de time de resposta rápida capaz de atender a todos os pacientes em risco; e (3) monitoramento dos indicadores relacionados à intervenção. Este trabalho empregou quatro ciclos semestrais (Planejar-Fazer-Estudar-Agir), com a finalidade de testar e ajustar a intervenção, entre janeiro de 2013 e dezembro de 2014.

**Resultados:**

Entre 2013 e 2014, o time de resposta rápida atendeu 2.296 pacientes. Houve redução não significante da mortalidade de 8,3% no ciclo 1, para 5,0% no ciclo 4; contudo, o número de óbitos permaneceu estável nos ciclos 3 e 4, com frequência de 5,2% e 5,0%, respectivamente. Com relação ao fluxo de pacientes e cuidados críticos continuados − uma premissa do time de resposta rápida −, houve decréscimo no tempo de espera por um leito na unidade de terapia intensiva, com diminuição de 45,9% para 19,0% na frequência de pacientes hospitalizados que não puderam ser imediatamente admitidos após a indicação (p < 0,001), representando melhora no fluxo de pacientes do hospital; ocorreu também aumento no reconhecimento de pacientes para cuidados paliativos, de 2,8% para 10,3% (p = 0,005).

**Conclusão:**

A implantação de um time de resposta rápida pode trazer benefícios nos contextos em que ocorrem restrições estruturais, como falta de leitos em unidades de terapia intensiva, porém há necessidade de alguns ajustes.

## INTRODUCTION

Considering concerns regarding lethality resulting from the relatively frequent occurrence of cardiopulmonary failure, the long-term experience with cardiopulmonary resuscitation (CPR),^(1)^ and evidence of the interference in these results of early identification of signs and care provided to the patient in noncritical units,^(2)^ rapid response teams (RRTs) have been widely implemented as an inpatient care quality improvement intervention in developed countries.^(3-5)^ These units involve specialized multidisciplinary teams responsible for the prompt evaluation, screening and treatment of patients with signs of deterioration in general wards.^(2)^

In middle and low-income countries, however, the implementation of RRTs has been more sporadic with few studies published.^(6-11)^ A more unfavorable economic context is likely to affect necessary investments in acquiring equipment and hiring staff.^(6,9)^ More hierarchical professional relations and cultural norms may constitute barriers to teamwork, a fundamental attribute of the intervention.^(12,13)^ Additionally, in these countries, healthcare monitoring is uncommon with data collection seen as a costly and time-consuming task by frontline professionals.^(6,13,14)^ This mentality represents a challenge in any healthcare quality improvement effort. The RRT call system design still requires more careful planning to account for necessary adjustments to the context and resources available.

The implementation of RRTs in Latin American countries, for example, may be especially relevant considering the scarcity of intensive care unit (ICU) beds in the face of systemic financial, structural and political problems,^(6,9,14,15)^ which contrasts to ICU overcrowding in developed countries generally associated with population aging.^(16,17)^ While in studies conducted in England,^(18)^ France,^(19)^ and Hong Kong,^(20)^ estimates of late ICU admissions are 32.6%, 37.6% and 37.8%, respectively. In Brazil, the late ICU admission rate is 68.8%,^(14)^ with an expected increase of 1.5% in the risk of death for each one-hour increase in the waiting time to ICU admission.^(14)^ Moreover, the implementation of RRT could allow for a reduction in nonplanned ICU admissions and increase in access to more specialized and quality improved health care as a result of better attendance planning.

The RRT intervention focused on in this study was designed to facilitate earlier identification of aggravation signs among inpatients on general wards in a large Brazilian general hospital and was inspired by successful results in the implementation of RRT in the international scene.

This study seeks to describe the RRT implementation experience, presenting its effects on the quality of critical care on general wards and indicating lessons that may be useful in building knowledge about RRT implementation in similar contexts.

## METHODS

The quality improvement effort reported here occurred in a large tertiary nonprofit hospital that serves as a referral site for a macroregion involving more than 94 municipalities and 1,500,000 inhabitants in the State of Minas Gerais, Brazil. The hospital has more than 70% of its 508 beds reserved for the *Sistema* Único *de Saúde* (SUS) (Brazilian universal public health system), and its 40 ICU beds are distributed into general, surgical and cardiological ICU for adults. In addition, the hospital also has a pediatric ICU.

Given that the ICU beds are continuously completely occupied at this hospital and a large number of patients are in queue for critical care, the perception about the occurrence among these patients of avoidable adverse events, including deaths, emerged in 2011, despite the absence of systematic measurements. The hospital administration had been changed, and other healthcare quality improvement interventions were implemented.

As is common in low and middle-income countries, the hospital was not used to monitor process and outcome indicators or to deal systematically with quality improvement initiatives. Although the intervention was initially motivated strictly by perceptions, it led to an interest in following the changes produced overtime and was assessed together with the implementation as a learning process itself.

This study was approved by the Research Ethics Committee of the hospital where the study was conducted (CAAE 45752315.4.0000.5147).

### Measures and analysis

Despite the weakness of having no baseline measurements before the implementation of the intervention, the analysis incorporated the monitoring of some metrics alongside its implementation and adjustments. In this sense, process and outcome indicators regarding the RRT actions were recorded between January 2013 and December 2014.

The measurements and analyses were initially focused on the following metrics: number of RRT attendances by hospital admissions; RRT response time; RRT attendance outcomes on general wards; hospital mortality; and the RRT call mode. Rapid response team attendance outcomes accounted for the possibilities of remaining on general ward, being transferred to ICU or surgical center, and death. In the case of transference to ICU, time required was computed. The analyses included all adult nonobstetric patients. Palliative care patients were excluded. With the emergence of new objectives over time, the following metrics were added: frequency of patients with indication for ICU admission by RRT and waiting for a vacancy on general wards; and trigger frequency for patients with cardiopulmonary arrest (CPA).

With the exception of hospital mortality, data were collected and loaded in a specific database of RRT attendances. Hospital mortality was obtained from variables routinely measured accounting for admissions and deaths, excluding patients from obstetrics and pediatrics.

For each semester of the intervention implementation, we obtained the total number of RRT attendances per 100 admissions, the mean and standard deviation for the continuous variable 'response time', and absolute and relative frequencies for different categorical variables. We also compared the categorical variables' distributions among semesters based on the chi-squared test considering a significance level of 0.05. In all the cases, we used Statistical Package for Social Science (SPSS) software, version 21.

Additionally, we employed SAS^®^ version 9.4 to build a statistical control chart on a monthly basis for 'ICU indication waiting for a vacancy' from July 2013 when the metric was incorporated in the project.

### Intervention

In general terms, the intervention consisted of three major components: a tool to detect aggravation of clinical conditions among inpatients on general wards, identifying those in need of specialized care or ICU admission; the structuring of an RRT to attend to all patients at risk; and the monitoring of indicators as described above to capture changes introduced and support adjustments in the intervention itself.

The tool to detect aggravation of clinical conditions among inpatients on general wards was elaborated by the hospital quality improvement group and corresponds to a score table indicating the need of calling the RRT - "trigger table" (Table 1). The trigger table was structured by the improvement team composed of physicians after an extensive literature review. The choice of different items on the table and the necessary score for its activation were based on the review and professional experience. The development of the trigger table was aimed at assisting healthcare professionals, especially nurses, in the early identification of signs of aggravation and generating adequate activation of the RRT based on the score (≥ 3) obtained by routine examination. The aggravation signs included in the trigger table were heart rate, systolic blood pressure, respiratory rate, urinary debt, central nervous system response, oxygen saturation, ventilator support, chest pain and bleeding.

**Table 1:** Table for calling the rapid response team (trigger table)

	Score						
	3	2	1	0	1	2	3
Heart rate	No pulse	< 40	41 - 50	51 - 100	101 - 110	111 - 130	> 130
Systolic blood pressure	Inaudible	70 80	90	100 170	180 190	200 210 220	> 220
Respiratory rate		< 8	8 - 11	12 - 20	21 - 25	26 - 30	> 30
Urinary debt/mL (last 4 hours)	< 80	80 - 120	121 - 200		> 800		
Central nervous system			Confused	Awake and responsive	Responds only to verbal command	Responds only to painful stimulus	No response to stimulus
Oxygen saturation	< 85%	86% - 89%	90% - 94%	> 95%			
Ventilator support	BiPAP/ CPAP	High flow	Oxygen therapy				
Trigger RRT if 3 points or more							
Chest pain	Trigger RRT						
Bleeding							

BiPAP - bilevel positive-pressure airway pressure; CPAP - continuous positive airway pressure; RRT - rapid response team.

The RRT involved a dedicated critical care specialist nurse and an available hospitalist physician called by the nurse when necessary. In the second year of the project, a dedicated physiotherapist was also included in the team.

During the first semester, which was treated as a pilot phase in the project, only one specialist nurse was hired, covering business hours. From the second semester, the RRT coverage was extended to the full 24-hour daily period, requiring the maintenance of at least four nurses working on a 12-hour shift to 36-hour rest basis. The physiotherapist covered only business hours.

In contrast to other RRT initiatives in which physicians are responsible for the first attendance after calls, this initiative opted to hire dedicated nurses and make them responsible for the immediate attendance by the RRT to reduce costs in the context of scarce financial resources. A physician was always available but not exclusively dedicated to the RRT; the physician was only called in more critical situations.

Managerial supervision of the RRT actions and performance was a duty of a member of the quality improvement group.

A specific group of professionals with a background in public health was in charge of the monitoring component, proposing the variables/indicators (presented above) and designing a specific form to be fulfilled by the RRT at each trigger.

The intervention aimed at providing immediate care to emergencies that occurred in inpatients on general wards to prevent clinical aggravations and avoidable deaths. During the project development, the intervention and its implementation were adjusted based on the results observed as described below.

### Evaluation methods

This work implemented Edwards Deming's Plan-Do-Study-Act (PDSA) cycles to test and adjust the intervention^(21)^ as foreseen and planned by the quality improvement group. Four six-month PDSA cycles were performed, including reliable data collection and analysis. Table 2 systematizes actions that were recommended at the end of each cycle to be executed in the next cycle.

**Table 2:** Improvement actions conducted from specific targeting metrics at the end of each cycle

Cycle	Period	Goal(s)	Actions for the next cycle of PDSA
Initial planning	Before 2013	Implement the RRT	Literature review and preparation of the table for calling the RRT (trigger table) Preparation and dissemination of an institutional protocol to call the RRT Hiring experienced nurse for exclusive and immediate action in urgency cases of general wards Hiring of professionals specialized in data analysis to monitor the process Specific form structuring with variables of interest to the results analysis Elaboration of agenda for presentation and discussion of results
1	1^st^ semester/2013	Increase care through the RRT Greater standardization of RRT care	Hiring experienced nurses for dedicated and immediate action in emergency cases on general wards for 24 hours Team training per the institutional protocol Acquisition of corporate cellular handsets for RRT members
2	2^nd^ semester/2013	Adequate intensive support in noncritical units	Acquisition of necessary equipment for critical patient care Standardization of emergency carts and regular internal audit by RRT members Bed management Acquisition of a specific outfit to identify and highlight the RRT
3	1^st^ semester/2014	Reduced RRT triggers due to CPA	Continuing education on intensive care were provided to the RRT (ACLS course) Hiring physiotherapist for the RRT
4	2^nd^ semester/2014	Sustaining the RRT Reduced mortality	New dissemination of the Table for calling the RRT (trigger table) to general ward teams; Structuring of per color trigger criteria, related to gravity Establishment of a team for specialized care to patients in palliative care.

PDSA - Plan-Do-Study-Act; RRT - rapid response team; CPA - cardiopulmonary arrest; ACLS - Advanced Cardiovascular Life Support.

### Cycle 1

An initial project pilot phase occurred in the first semester of 2013 when a critical care specialist nurse worked in the emergency care of nonintensive units during business hours Monday through Friday from 07h00 through 16h00. As of January 2013, RRT was called by a specific telephone extension in cases of score ≥ 3 in the evaluation of the trigger table, which was duly described and disseminated through institutional protocol. At the end of cycle 1, meetings for dissemination and discussion of the results were held. The meetings aimed to guide improvement actions and establish goals for a new cycle in the next six months with the participation of members of the quality improvement group, the RRT, the data-monitoring group and hospital managers.

### Cycle 2

The initial plan of providing a 24-hour daily RRT coverage was implemented in July 2013 with three additional nurses hired for exclusive and immediate action in emergencies on general wards of the hospital. Another adjustment introduced was the incorporation of corporate cellular handsets for the RRT members to facilitate rapid and direct communication. Additionally, monitoring of the variable 'ICU indication waiting for a vacancy' was initiated.

### Cycle 3

This cycle was characterized by the implementation of a bed management system, acquisition of necessary equipment for critical patient care in noncritical units, and standardization of emergency carts. The variable indicating 'trigger due to CPA' was incorporated.

### Cycle 4

The actions were aimed at sustaining the RRT and reduced mortality. Continuing education on intensive care to RRT (Advanced Cardiovascular Life Support course - ACLS) and new dissemination of the trigger table to nonintensive care unit teams were provided.

The Standards for Quality Improvement Reporting Excellence (SQUIRE) framework provided criteria for the publication of this manuscript.^(22)^

## RESULTS

**Figure 1:**
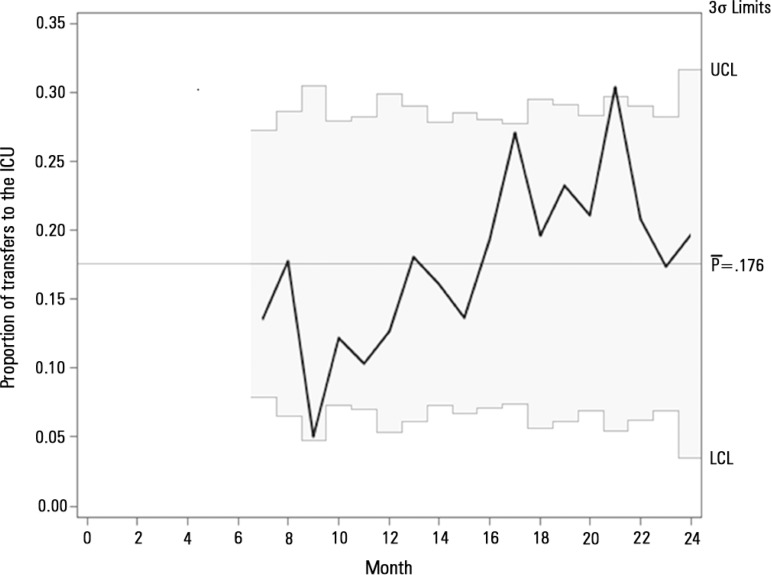
Statistical control chart for transferences to the intensive care unit after rapid response team attendance. The statistical control chart shows the proportion of patients referred to intensive care unit by the rapid response team who was admitted immediately. ICU - intensive care unit; UCL - upper control limit; LCL - lower control limit.

From January 2013 to December 2014, the RRT attended to 2,296 patients. In the pilot period of cycle 1, 3.1 attendances were performed per 100 hospital admissions. In the other cycles, when the RRT was fully operational, the number was always greater than 9.0. Rapid response teams mean response time upon trigger was less than 5 minutes in all cycles. Regarding the RRT attendance outcomes of general wards at all cycles, most patients had clinical conditions to remain in the unit following RRT's intervention. Notwithstanding, the initial death rate decrease on general wards was reduced from 8.3% to 5.1% from cycle 1 to cycle 2, and death rates remained stable in cycles 3 and 4, with frequencies of 5.2% and 5.0%, respectively. Hospital mortality also remained stable. There was a significant increase in recognition of palliative care patients from 2.8% in cycle 1 to 10.3% in cycle 4 (p = 0.005). Regarding RRT call mode, there was a significant reduction both in the number of nonstandardized forms (21.7% to 2.9% between cycle 1 and 4) and lack of data filling in the form by the team (p = 0.000). Statistical significance was also observed for the decreased frequency of inpatients with ICU indication who needed to wait for a vacancy on general wards from 45.9% in cycle 2 to 19.0% in cycle 4 (p = 0.000). The increase in the proportion of patients transferred to the ICU immediately after the RRT attendance can be observed in the statistical control chart (Figure 1). The number of patients with trigger due to CPA was reduced from 5.9% in cycle 3 to 4.9% in cycle 4, but this difference was not statistically significant. Table 3 shows all results for each metric by cycle.

**Table 3:** Metrics results by cycle

Metric	2013				2014				p value
	Cycle 1		Cycle 2		Cycle 3		Cycle 4		
	%	n*/n†	%	n*/n†	%	n*/n†	%	n*/n†	
RRT attendance per 100 hospital admissions (N)	3.1		9.9		10.6		9.2		
Response time (min)	4.7	(± 3.7)	4.9	(±6.7)	4.4	(±4.0)	4.5	(±4.6)	
RRT attendance outcomes									
Remaining in unit	64.2	131/204	70.7	454/642	68.9	462/671	66.3	387/584	
ICU indication	25.5	52/204	23.1	148/642	25.8	173/671	27.1	158/584	
Surgical unit indication	2.0	4/204	1.1	7/642	0.1	1/671	1.6	10/584	
Death	8.3	17/204	5.1	33/642	5.2	35/671	5.0	29/584	0.060
Data unavailable	1.4	3/207	1.5	10/652	0.0	0	0.0	0	
Triggers for patients in palliative care	2.8	6/213	7.8	55/707	7.4	54/725	10.3	67/651	0.005
Hospital mortality	3.5		3.6		3.2		3.5		
RRT call system									
Specific extension	62.3	43/69	67.3	454/674	71.1	501/705	71.3	461/647	
Medical team	13.0	9/69	11.3	76/674	8.6	61/705	9.4	61/647	
Active search	2.9	2/69	12.8	86/674	13.8	97/705	16.4	106/647	
Other nonstandardized devices	21.7	15/69	8.6	58/674	6.5	46/705	2.9	19/647	0.000
Data unavailable	67.6	144/213	4.7	33/707	2.8	20/725	0.6	4/651	
ICU indication waiting for a vacancy	-	-	45.9	68/148	26.0	45/173	19.0	30/158	0.000
Triggers due to cardiopulmonary arrest	-	-	-	-	5.9	43/725	4.9	32/651	0.480

RRT - rapid response team; ICU - intensive care unit.

* Number of triggers by collected variable;

† Total number of triggers.

From the initial planning for RRT implementation, different improvement actions were conducted at the end of each cycle, considering specific directional metrics according to the PDSA (Table 2). At the end of the first semester of 2013, completing cycle 1, the number of attendances by the pilot's RRT and the low response time showed the possibility of expanding this service, contributing to the recruitment of other experienced nurses for dedicated and immediate action in cases of emergency on general wards for 24 hours. In addition, the high number of calls through nonstandardized devices (21.7%) highlighted the need for greater standardization of care, giving rise to team-training actions (RRT and nonintensive units care team) according to institutional protocol. Corporate cellphones handsets were also acquired to facilitate communication in the trigger action when specialist nurses were more easily located by the specific extension.

On completion of the second cycle, 45.9% of the patients with ICU indication were not admitted immediately and remained on general wards waiting for a vacancy; thus, the need for adequate intensive support was also noted in these open units. Thereafter, actions taken included the purchase of equipment, standardization of emergency carts and bed management. The acquisition of specific outfits for the RRT providing its identification and distinction was included as a new action to stimulate the culture of this intervention in the institution.

At the end of the third cycle, when stabilization of deaths on general wards was observed and the frequency of trigger due to CPA was 5.9%, two actions were implemented to improve care of critical patients on general wards: continuing education on intensive care was provided to the RRT (Advanced Cardiovascular Life Support course - ACLS) and a physiotherapist was hired for joint action with the team. The stabilized number of deaths among patients treated by the RRT and hospital mortality observed at the end of cycle 4 together with an increasing number of patients in palliative care alerted to the need for future actions aimed at the new dissemination of the Trigger Table on general wards, structuring of new severity-related criteria and establishment of a team for specialized care for patients in palliative care.

## DISCUSSION

From the implementation of an RRT led by nurses, it was possible to observe the following: a reduction in mortality albeit not statistically significant by simultaneous comparison of all PDSA cycles; a reduction of inpatients who could not be admitted to the ICU immediately after indication, which represents a reduction in waiting times for an ICU and improved patient flow in the hospital; and an increase in recognition of palliative care patients, which may result in better resource utilization of scarce ICU beds.

The patient mortality was reduced from 8.3% to 5.1% between cycles 1 and 2 followed by stabilization in cycles 3 (5.2%) and 4 (5.0%). The overall frequency was low (5.9%), contrasting with estimates from other studies ranging from 10.6% to 56.7%^(6,23,24)^ regardless of whether palliative care patients were excluded. Despite the stabilization of mortality, patient flow through the hospital system improved.

In the evaluation of cycle 2, 45.9% of inpatients with indication for ICU care were not admitted immediately, and this result decreased significantly to 26.0% and 19.0% in cycles 3 and 4, respectively. The high initial figure is consistent with that observed in Brazil and other Latin American countries,^(25)^ where it is estimated that most patients with indication for ICU treatment are not immediately admitted.^(14)^ This delayed admission to the ICU can contribute to a lengthier hospital stay^(26)^ and increase the risk of death among these patients by up to five-fold^(27)^ with a progressive worsening of organic function and mortality at each waiting hour.^(13)^ The reduced number of patients awaiting a vacancy may be related to improvement-oriented actions over time, such as bed management. For effective care and intensive care delivery, even outside the ICU, equipment and standardized emergency carts were purchased and respiratory therapy provided. These actions relate to the idea of continuum care, which is fundamental for the premise of a rapid response system.^(28)^

The number of palliative care patients is associated with institutional characteristics of the hospital and a large proportion of elderly patients. The growing recognition of palliative care patients over time may indicate more team-family joint action with family members having more opportunity to interact with healthcare professionals about patients' prognoses. Implementation of RRT has been associated with an increase in do-not-resuscitate order placement.^(29)^

Recognizing the importance of tracking critical patients through shift handovers and even assuring beforehand the availability of beds in the ICU to receive them, the RRT's commitment to an active search of critical patients is an aspect to be highlighted and may be related to the hiring of exclusive professionals for the team. In 11.5% of the cases registered in the study period, patients were identified through an active search by the RRT. According to the protocol established, the specialist nurse should be always the first professional to be called in an RRT. However, this did not occur in 10.6% of the cases during the entire period, in which the hospitalist was called before followed by the nurse. On one hand, this finding may indicate noncompliance or lack of knowledge about the established standard. On the other hand, this finding may indicate the hospitalist's adherence to the RRT protocol, valuing the specialist nurse's role. Undoubtedly, this finding contrasts the strong hierarchical professional relations still dominant in developing countries^(12,13)^ and expresses a positive attitude in favor of teamwork.

The strategy of making nurses central in the RRT and responsible for the first attendance after calls is relevant in maintaining lower costs. The strategy was appreciated as more realistic and sustainable considering the limited financial resources.^(13)^ A study developed in a hospital in the United States focused on the experience of an RRT led by nurses but found no reduction in mortality after its implementation.^(30)^ On the other hand, the optimal composition of an RRT remains unknown in the literature, and no studies have compared clinical outcomes of medical emergency teams with those of nonphysicians-led RRTs.^(31)^

Also noteworthy is the observation of the substantial improvement in data fulfilling of the call system over the PDSA cycles. Despite the frequent use of routinely collected data to support planning and management in healthcare organizations, the task is often considered tedious by healthcare teams, requiring the unrelenting search for alternatives to the systematization of reliable data collection. The improvement of adherence to form fulfilling over time can be attributed to the team's inclusion in the discussion of results, leading to greater recognition of the importance of records.

In this work, response time between trigger and arrival of an RRT nurse at the patient's bed was always less than five minutes, but values may be questionable as they were collected by RRT members. Given the objective of rapid response with immediate action, prompt evaluation, screening and treatment of patients with signs of clinical deterioration, one could expect lower times to be recorded by the team itself. Despite the recognition of the need for technological resources for this measurement, its use was not possible in the hospital, requiring the adaptation of resources according to what local conditions allowed.

While metrics were established in the study initial monitoring plan, new metrics were added after the first cycle with limited comparative potential. The frequency of patients awaiting a vacancy for the ICU was perceived as important given the large proportion of patients in that situation at the end of cycle 1. The frequency of trigger due to CPA may subsequently indicate how early triggers and attendances are. Its measurement exclusively in cycles 3 and 4 compromised its meaning and ability to provide information. Studies evaluating RRT performance through CPA frequency report a reduction ranging from 17% to 50%,^(32,33)^ but the isolated consideration of the indicator may imply biases since CPA may not be frequent in the nonintensive care units but may occur in the ICUs after referral of the patient.

An important limitation of this work was related to lack of patients' clinical and sociodemographic profiles. This unknown information could potentially affect the analysis of deaths, allowing further considerations about mortality. Additionally, it is noteworthy that general hospital mortality could not be compared statistically between the different cycles because it results from numbers provided by the organization that are not part of the database used in the other analyses.

Another limitation was not accounting for a baseline period with measurements prior to the intervention, making comparisons impossible. Data monitoring was incorporated as a component of the intervention and involved systematic data collection. However, the process was initiated while other actions were already occurring. To some extent, it reveals certain voluntarism and inexperience of the quality improvement group at the beginning of the work, which is likely not rare among well-intentioned healthcare improvement groups moved by the desire to promote positive changes but lacking structured theory-based plans. However, if the limitation itself slightly compromised the capacity of more precisely evaluating the effects of the intervention, we still have evidence of amelioration of indicators. Moreover, lessons learned ratify the importance of more systematic approaches in healthcare quality improvement as proposed by Improvement Science.^(34)^

Despite the impact of RRT implementation in different locations,^(3-5)^ its effectiveness can be influenced by the characteristics of each organization.^(35)^ This intervention was performed in a developing country in an organization that seeks to modernize itself but coexists with conservative culture with many professionals resistant to change. The often-scarce resources may also serve as a barrier to implementation at times in addition to power disputes. In a healthcare organization where services provided by operators are complex, requiring a high degree of expertise and training in skills development, how power is distributed or the characteristics of who owns it can have a major influence on project development. However, the involvement and motivation of RRT professionals and those responsible for the analysis and dissemination of results as well as the support of some leaders may have been fundamental to the success of the implementation.

## CONCLUSION

This study allowed for the observation of effects of rapid response teams in attending emergencies on the general wards of a hospital over four six-month Plan-Do-Study-Act cycles. This policy constitutes a good strategy to follow the intervention due to the continuous search for improvement and the involvement of the team in the discussion of the results. The participation of different professionals in the half-yearly meetings allowed the alignment of different interests and provided greater practical feasibility to the actions intended from the concrete observations of weaknesses.

Although the results are not generalizable, this study highlights practical elements that should be considered in other similar efforts to implement a rapid response team. There was no further study of the context in which the intervention was developed, which poses the prospect of developing a future qualitative study with a view to understand the mechanisms of change of the intervention shown.
